# TikTok video as a health education source of information on heart failure in China: a content analysis

**DOI:** 10.3389/fpubh.2023.1315393

**Published:** 2023-12-11

**Authors:** Xun Gong, Bo Dong, Li Li, Danping Shen, Zhiyi Rong

**Affiliations:** Department of Cardiology and Cardiac Rehabilitation Center, Hunan Provincial People’s Hospital (The First Affiliated Hospital of Hunan Normal University), Changsha, China

**Keywords:** heart failure, health education, self-care, social media, TikTok, content quality

## Abstract

**Background:**

Heart failure (HF) is a complex and life-threatening syndrome associated with significant morbidity and mortality. While TikTok has gained popularity as a social media platform for sharing HF-related information, the quality of such content on TikTok remains unexplored.

**Methods:**

A cross-sectional analysis was conducted on TikTok videos related to HF in China. The sources of the videos were identified and analyzed. The content comprehensiveness of the videos was evaluated using six questions that covered definition, signs and symptoms, risk factors, evaluation, management, and outcomes. The reliability and quality of the videos were assessed using three standardized evaluation instruments: DISCERN, JAMA benchmarks, and the Global Quality Scale. Additionally, the correlation between video quality and video characteristics was further investigated.

**Results:**

Among the video sources, 92.2% were attributed to health professionals, while news agencies and non-profit organizations accounted for 5.7% and 2.1%, respectively. The content comprehensiveness score for the videos was 3.36 (SD 3.56), with news agencies receiving the highest scores of 4.06 (SD 3.31). The median DISCERN, JAMA, and GQS scores for all 141 videos were 26.50 (IQR 25.00–28.750), 2.00 (IQR 2.00–2.00), and 2.00 (IQR 2.00–2.00), respectively. Videos from health professionals had significantly higher JAMA scores compared to those from non-profit organizations (*P* < 0.01). Correlation analysis between video quality and video characteristics showed positive correlations between content comprehensiveness scores and video duration (*r* = 0.420, *P* < 0.001), number of comments (*r* = 0.195, *P* < 0.05), and number of shares (*r* = 0.174, *P* < 0.05). GQS scores were negatively or positively correlated with the number of days since upload (*r* = −0.212, *P* < 0.05) and video duration (*r* = 0.442, *P* < 0.001).

**Conclusion:**

The overall quality of the videos was found to be unsatisfactory, with variations in quality scores observed across different video sources. Content comprehensiveness was inadequate, the reliability and quality of the information presented in the videos was questionable. As TikTok continues to grow as a platform for health information, it is essential to prioritize accuracy and reliability to enhance patients’ self-care abilities and promote public health.

## Introduction

Heart failure (HF) is a multifaceted and life-threatening syndrome characterized by significant morbidity, mortality, impaired functional capacity, reduced quality of life, and high healthcare costs. The global prevalence of HF exceeds 64 million people, leading to a substantial burden on healthcare expenditures (1). In the United States, the estimated cost of HF in 2012 was $30.7 billion, with projections indicating a 127% increase to $69.8 billion by 2030, amounting to approximately $244 per adult (2). HF has also emerged as a significant public health concern in China, where the number of patients suffering from HF was 8.90 million (3). The overall crude prevalence and incidence of HF were 1.18% and 248 per 100,000 patient-years, respectively (4). The sheer magnitude of patients with HF in China, as the most populous nation globally, underscores the scale of this health issue. The rising prevalence of HF can be attributed to factors such as population aging, improved treatment and management of ischemic heart disease, and the availability of evidence-based therapies that extend the lives of HF patients (1). Consequently, there is a significant demand for medical care among patients with HF. However, the current scarcity of medical resources poses challenges in meeting the consultation and treatment needs of these individuals (5).

Seeking medical consultation and treatment can be a challenging and time-consuming process, involving several steps such as appointment registration, waiting for consultations, examinations, and treatments. The issue of long waiting times for inpatient and outpatient services is pervasive globally. For example, in the Spanish National Health System, the average waiting time to see a general practitioner is 3.36 days, whereas the waiting time for specialist consultations is 88.03 days (6). Another study from China revealed that patients’ satisfaction with outpatient waiting times was only 28.8%, with higher-level hospitals receiving lower satisfaction ratings (7). Additionally, even when patients do interact with medical staff, the busy schedules of healthcare professionals often limit the attention given to detailed information consultation and health education for patients (8). Following completion of outpatient treatment or discharge from the inpatient department, patients with HF require regular follow-up to modify their treatment plans, such as adjusting diuretic dosages in response to symptoms, weight, or urine volume (9). However, due to time constraints faced by medical staff, providing adequate follow-up care becomes challenging, resulting in patients frequently experiencing worsening symptoms that necessitate readmission. In China, hospitalized HF patients typically undergo an average hospital stay of 10 days (ranging from 7 to 15 days) per admission, accompanied by a notable hospital mortality rate of 4.1 ± 0.3% (10).

In this scenario, patients must acquire self-care knowledge and skills to minimize readmissions caused by worsening symptoms, often seeking information through alternative channels. In China, the information needs of HF patients primarily encompass risk factors, symptom management, diagnosis, and lifestyle adjustment (11). However, given the limitations of the existing medical system in meeting the growing demand for HF information among patients, innovative approaches are needed to bridge this gap. In the present era, with advancements in smartphones and information technology, an increasing number of short video platforms are offering relevant disease information to patients in need (12). Accessing disease information through these platforms offers significant advantages. Firstly, acquiring information is convenient and time-saving, eliminating the need for travel or facing time restrictions associated with conventional medical services. Patients can access the information repeatedly at their convenience. Secondly, obtaining information through short videos is cost-effective. These videos are generally provided free of charge and require minimal network traffic and electricity fees. Conversely, seeking medical treatment at a hospital incurs substantial expenses related to medical services, transportation, accommodation, catering, and time costs (13). Thirdly, this method of information acquisition helps dispel doubts arising from relying on a single information source. Unlike hospital visits, where patients can consult only one doctor, short video platforms host multiple uploaders who provide diverse opinions and information. This indirect verification of information obtained from hospital treatment mitigates the distrust between doctors and patients caused by limited information sources (14). Lastly, this mode of information acquisition effectively protects patient privacy. Seeking medical treatment at a hospital carries potential privacy risks (15), whereas watching short videos does not raise similar concerns and reduces the need for physical contact with others (16). Consequently, short video platforms have become crucial sources of disease information for the general public. TikTok, as the world’s most widely used short video platform, also offers numerous videos related to HF (17). Nonetheless, the quality of disease-related videos on TikTok varies greatly, posing challenges for patients in identifying reliable information and increasing the risk of being misled. For example, videos on TikTok related to chronic obstructive pulmonary disease, diabetes, gallstones, and inflammatory bowel disease generally exhibit low reliability and insufficient treatment information (18–21). Given the greater severity and complexity of HF compared to the aforementioned diseases, there is a need for higher-quality HF-related videos. Nonetheless, the quality of HF-related videos on TikTok remains unexplored, highlighting the critical need for their assessment. Hence, the primary objective of this study is to evaluate the quality of HF-related videos on TikTok and provide accurate recommendations for patients and video producers.

## Methods

### Search strategy and data extraction

**Figure 1:**
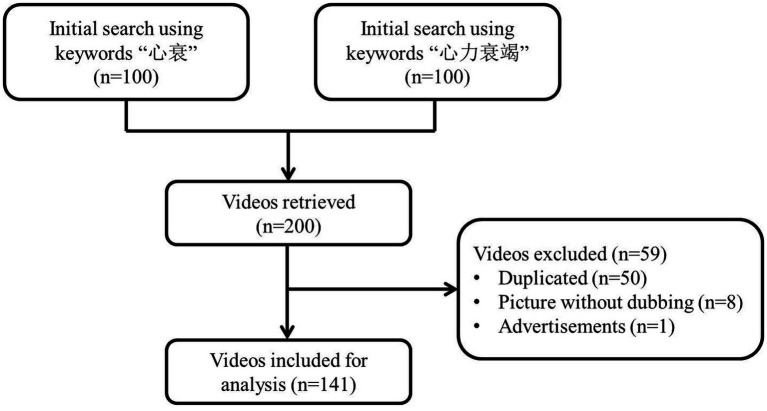
Search strategy and video screening procedure.

To retrieve relevant TikTok videos on HF, we searched using two Chinese words: “心衰” (short name for HF) and “心力衰竭” (full name for HF). TikTok offers three sorting options for search results: “overall ranking,” “most recent,” and “most likes.” The default sorting mode recommended by TikTok is the overall ranking, which includes the other two modes. As most users use the default setting, we retrieved the top 100 videos for each keyword on August 6, 2023, using the overall ranking mode. This resulted in a total of 200 videos. We selected a threshold of 100 videos for two reasons. First, TikTok’s search function considers topic relevance, and the most relevant HF videos appear at the top of the result list. Finding relevant videos becomes challenging when the results exceed 100. Second, most general health consumers follow the principle of “least effort” when seeking online information, typically viewing the top search results rather than exploring extensively (22). To select the most relevant videos, we removed duplicates (*n* = 50) and videos that were only pictures without dubbing (*n* = 8), or advertisements (*n* = 1). Finally, we had a total of 141 videos for data analysis (Figure 1). We used Microsoft Excel to extract and code basic information from each video, including the video description, upload date, duration (in seconds), and number of likes, comments, collections, and shares.

### Classification of videos

The videos were categorized into the following sources (18): (1) Health professionals, (2) News agencies (such as network media, newspapers, TV stations, and radio stations), (3) Nonprofit organizations, and (4) For-profit organizations. This classification allows for grouping videos with similar content while distinguishing those with different content.

### Evaluating methodologies

#### Assessment of video content, reliability, and quality

The content, reliability, and quality of the videos were evaluated using scoring methods. We employed six questions from Goobie et al. (23) to assess the video content, focusing on the coverage of disease definition, signs and symptoms, risk factors, evaluation, management, and outcomes. Each aspect was scored on a three-point scale: not addressed (0 points), partially addressed (1 point), and sufficiently addressed (2 points). The DISCERN instrument (24, 25) was utilized to evaluate content reliability, considering the reliability of the videos, quality of treatment choices, and overall information quality. The instrument comprises 16 questions, with responses rated on a 5-point scale (1 = poor, 5 = excellent). These questions are categorized into three sections. The initial 8 questions assess the publication’s reliability, examining clarity, relevance, balance, and fairness of its objectives. Scores in this section gauge the publication’s suitability as a dependable resource for specific disease treatment methodologies. The second segment includes 7 questions delving into treatment details, evaluating if the publication delineates each treatment’s effects and elucidates associated risks and benefits. Scores in this section signify the quality of information in the publication concerning treatment choices, encompassing self-care. The concluding section, built on preceding inquiries, features a single question prompting users to evaluate the overall publication quality as an informational source on treatment choices (19). The DISCERN instrument has undergone extensive validation and is widely used for evaluating health-related content on various video-sharing platforms, including YouTube, TikTok, Kwai, and Bilibili (18, 19, 21, 23, 26). Additionally, we used the JAMA benchmark criteria (27, 28) to assess the reliability of video sources on a scale of 0 to 4. The criteria comprise four distinct components, and each component is allocated a score of 1. A total score of 4 signifies excellent quality, whereas a score of 0 indicates low quality. To evaluate the overall quality of the videos, we employed the Global Quality Score (GQS). The instrument assesses the quality and quantity of information, along with the value of the information source for lay users. The GQS is a commonly used 5-point Likert scale ranging from 1 (poor quality) to 5 (excellent quality) for the assessment of internet videos (20, 29, 30). The detailed information for DISCERN, JAMA Benchmark Criteria, and Global Quality Scale are available online as Supplementary material.

#### Evaluation procedure

To minimize bias introduced by personalized recommendation algorithms, a new TikTok account was created and used for the evaluation. Personalized recommendation function of TikTok was disabled to eliminate differential content recommendations caused by user habits. Evaluation tasks were carried out by two qualified physicians (XG and ZR) from the Division of Cardiology in a tertiary teaching hospital. XG was a specialist in cardiology with 13 years of experience in the field, including 5 years in cardiac rehabilitation. ZR was also a cardiology specialist with 24 years of experience, including 17 years in cardiovascular intervention and electrophysiology. All videos were viewed without performing actions such as downloading, liking, commenting, collecting, or sharing. Before scoring the videos, the two raters familiarized themselves with management guidelines from the American Heart Association (AHA) and the European Society of Cardiology (ESC) (31, 32), as well as the official scoring instructions for DISCERN, JAMA, and GQS. They then discussed and made necessary adjustments to operationalize the evaluation tools for video-based content. Each video was independently evaluated by the two raters, followed by a discussion and resolution of any inconsistencies. After reaching a consensus on video quality based on scoring the first 20 videos, the raters proceeded to independently complete the remaining scoring and calculate the average values.

### Ethical considerations

The present study did not involve the utilization of clinical data, human specimens, or laboratory animals. All the information utilized in this study was acquired solely from publicly accessible TikTok videos, ensuring the preservation of personal privacy. Additionally, the study did not involve any direct interaction with users, eliminating the need for ethics review or trial registration.

### Statistical analyses

Statistical analysis was performed using SPSS software (version 27.0; IBM), and data visualization was conducted using R software (version 4.3.1; R Foundation for Statistical Computing). Cohen’s kappa coefficients were calculated to assess interrater reliability, with values greater than 0.6 indicating good interrater reliability. Group comparisons were analyzed using the Kruskal-Wallis H test. Spearman correlation analysis was used to evaluate relationships between quantitative variables. A significance level of *P* < 0.05 was considered statistically significant.

## Results

### Video characteristics

After applying the inclusion and exclusion criteria, we selected a total of 141 videos for further data extraction and analysis (Figure 1). Based on the uploaders’ identities, the 141 videos were categorized into four groups: health professionals, news agencies, nonprofit organizations, and for-profit organizations. Out of the 141 videos, 130 were uploaded by health professionals (92.2%), 8 were uploaded by news agencies (5.7%), 3 were uploaded by nonprofit organizations (2.1%), and none were uploaded by for-profit organizations (0.0%) (Table 1). Among all the included videos, the median time since upload was 318 days (IQR 9–576), the median duration of the videos was 78 seconds (IQR 51–111), the median number of likes received was 1,107 (IQR 430–3,430), the median number of comments received was 81 (IQR 33–266), the median number of collections received was 223 (IQR 93–640), and the median number of shares received was 238 (IQR 105–818). Among the three groups, videos uploaded by health professionals had the shortest duration time, but they received more likes (median 1,176, IQR 430–3,684) and comments (median 83, IQR 33–270). On the other hand, videos uploaded by news agencies received more collections (median 242, IQR 79.5–3,357) and shares (median 815.5, IQR 274–6150.5) (Table 2).

**Table 1:** Characteristics of the videos across sources.

Source	Description	Videos, *n* (%)
Health professionals	Individuals who describe themselves as health professionals	130 (92.2)
News agencies	Organizations providing news services	8 (5.7)
Nonprofit organizations	Organizations or hospitals operating in the public sector	3 (2.1)
For-profit organizations	Private sector organizations	0 (0.0)

**Table 2:** Characteristics of the videos across sources (median numbers).

Source of videos	Days since upload, median (IQR)	Video duration (seconds), median (IQR)	Number of likes, median (IQR)	Number of comments, median (IQR)	Number of collections, median (IQR)	Number of shares, median (IQR)
Health professionals	314 (91,571)	72 (50,104)	1,176 (430,3,684)	83 (33,270)	226 (94,640)	213.5 (95,811)
News agencies	712.5 (378.5,1153.5)	147 (98.5,191)	857 (641.5,27,633)	69 (40,2,254)	242 (79.5,3,357)	815.5 (274,6150.5)
Nonprofit organizations	471 (380,544.5)	90 (79.5,93.5)	103 (71.5,1128.5)	29 (17,57)	43 (34.5,171)	49 (44,146.5)
Overall	318 (9,576)	78 (51,111)	1,107 (430,3,430)	81 (33,266)	223 (93,640)	238 (105,818)

Regarding the video presentation type, out of the 141 videos, 128 were narrative/outpatient shooting (90.8%), 2 were storytelling (1.4%), 2 were animated cartoons (1.4%), 8 were TV interview clips (5.7%), and 1 was dubbing pictures (0.7%) (Table 3).

**Table 3:** Video presentation type.

Type	Description	Videos, *n* (%)
Narrative or outpatient shooting	Medical videos shot in narrative tone, often showcasing real-life medical situations and outpatient procedures	128 (90.8)
Storytelling	Medical videos that effectively communicate information through a narrative structure, engaging viewers with a compelling storyline and characters	2 (1.4)
Cartoons	Animated medical videos that utilize illustrations or computer-generated imagery to educate and entertain viewers about medical topics	2 (1.4)
TV interview clips	Medical videos featuring segments or clips from television interviews with experts, providing insights and information on various medical subjects	8 (5.7)
Dubbing pictures	Medical videos where recorded audio, such as explanations or commentary, is synchronized with still images or slides to enhance understanding and engagement	1 (0.7)

### Information content comprehensiveness

**Figure 2:**
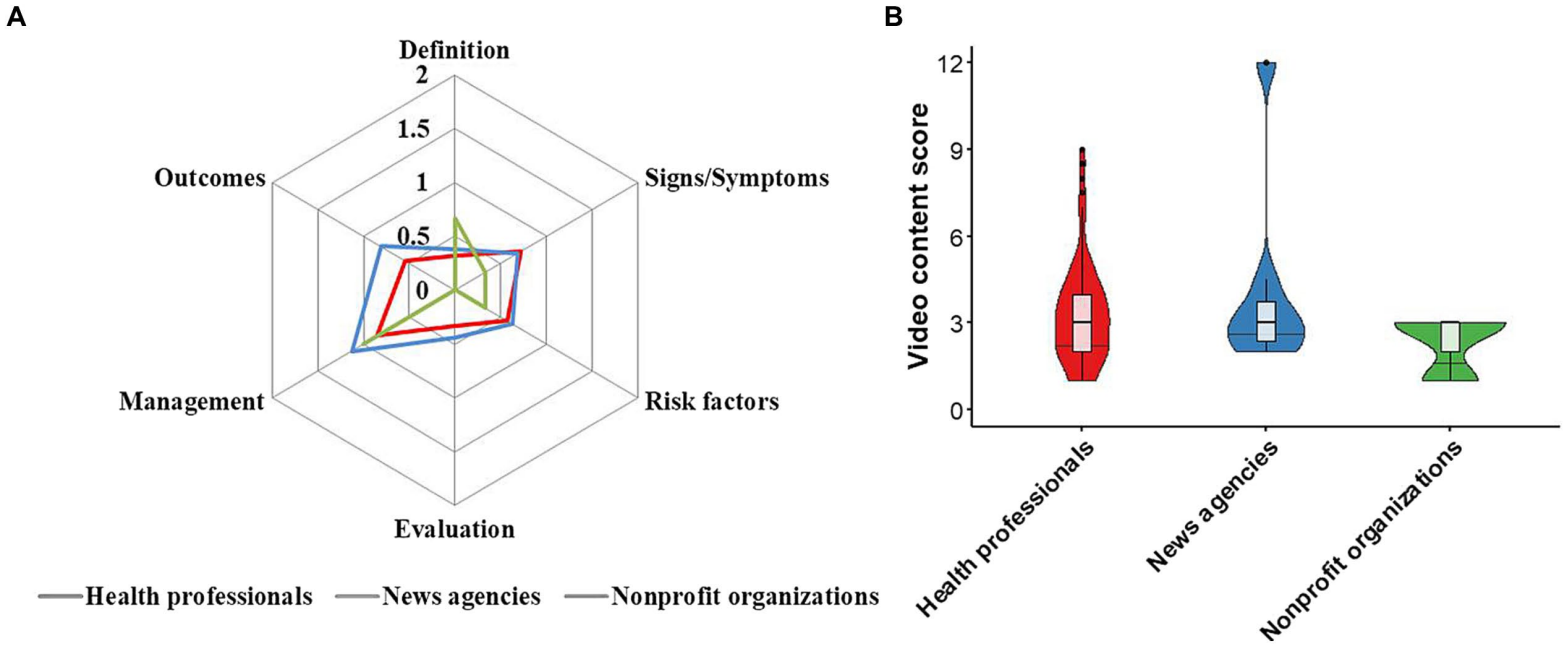
Comparison of content comprehensiveness between sources. **(A)** Radar charts showing the scores of content comprehensiveness among videos from different sources. **(B)** Violin plots showing the total content scores among videos from different sources.

The videos covered the six predefined content areas to varying degrees (Table 4). The mean scores for the six predefined content areas, namely definition, signs/symptoms, risk factors, evaluation, management, and outcomes, were 0.33 (SD 0.38), 0.71 (SD 0.49), 0.57 (SD 0.39), 0.33 (SD 0.27), 0.87 (SD 0.32), and 0.55 (SD 0.40), respectively (Table 4; Figure 2A). There was no significant difference in the total scores of content comprehensiveness among the three groups (*P* > 0.05) (Figure 2B).

**Table 4:** Comprehensiveness of video content.

Source of videos	Definition, mean (SD)	Signs/Symptoms, mean (SD)	Risk factors, mean (SD)	Evaluation, mean (SD)	Management, mean (SD)	Outcomes, mean (SD)	Total, mean (SD)
Health professionals	0.32 (0.60)	0.72 (0.70)	0.57 (0.62)	0.33 (0.51)	0.85 (0.58)	0.55 (0.63)	3.34 (1.79)
News agencies	0.38 (0.74)	0.69 (0.80)	0.63 (0.74)	0.44 (0.73)	1.13 (0.35)	0.81 (0.65)	4.06 (3.31)
Nonprofit organizations	0.67 (1.15)	0.33 (0.58)	0.33 (0.58)	0.00 (0.00)	1.00 (0.00)	0.00 (0.00)	2.33 (1.15)
Overall	0.33 (0.38)	0.71 (0.49)	0.57 (0.39)	0.33 (0.27)	0.87 (0.32)	0.55 (0.40)	3.36 (3.56)

It was noted that due to the majority of videos being uploaded by health professionals, variations in content may arise from their diverse professional backgrounds. Consequently, we categorized health professionals based on their specialties to assess the comprehensiveness of video content. The health professionals were classified into four types: 107 cardiologists, 12 cardiothoracic surgeons, 3 general practitioners, and 8 doctors from other specialties (including neurology, pain medicine, respiratory and critical care medicine, and gastroenterology and metabolic surgery). The mean total scores for video content comprehensiveness among these four types of health professionals were 3.25 (SD, 1.66), 4.25 (SD, 2.6), 4.50 (SD, 3.5), and 2.81 (SD, 0.75) respectively (Table 5).

**Table 5:** Comprehensiveness of video content across different health professionals’ types.

Source of videos	Definition, mean (SD)	Signs/Symptoms, mean (SD)	Risk factors, mean (SD)	Evaluation, mean (SD)	Management, mean (SD)	Outcomes, mean (SD)	Total, mean (SD)
Cardiologists (*n* = 107)	0.32 (0.57)	0.69 (0.71)	0.58 (0.62)	0.30 (0.49)	0.83 (0.59)	0.54 (0.59)	3.25 (1.66)
Cardiothoracic Surgeons (*n* = 12)	0.50 (0.80)	0.83 (0.72)	0.79 (0.66)	0.54 (0.66)	0.83 (0.72)	0.75 (0.87)	4.25 (2.6)
General practitioners (*n* = 3)	0.67 (1.15)	1.33 (0.58)	0.33 (0.58)	0.33 (0.58)	1.17 (0.29)	0.67 (1.15)	4.50 (3.5)
Others (*n* = 8)	0.00 (0.00)	0.75 (0.46)	0.25 (0.46)	0.44 (0.50)	1.00 (0.00)	0.38 (0.52)	2.81 (0.75)

### Information reliability and quality

**Figure 3:**
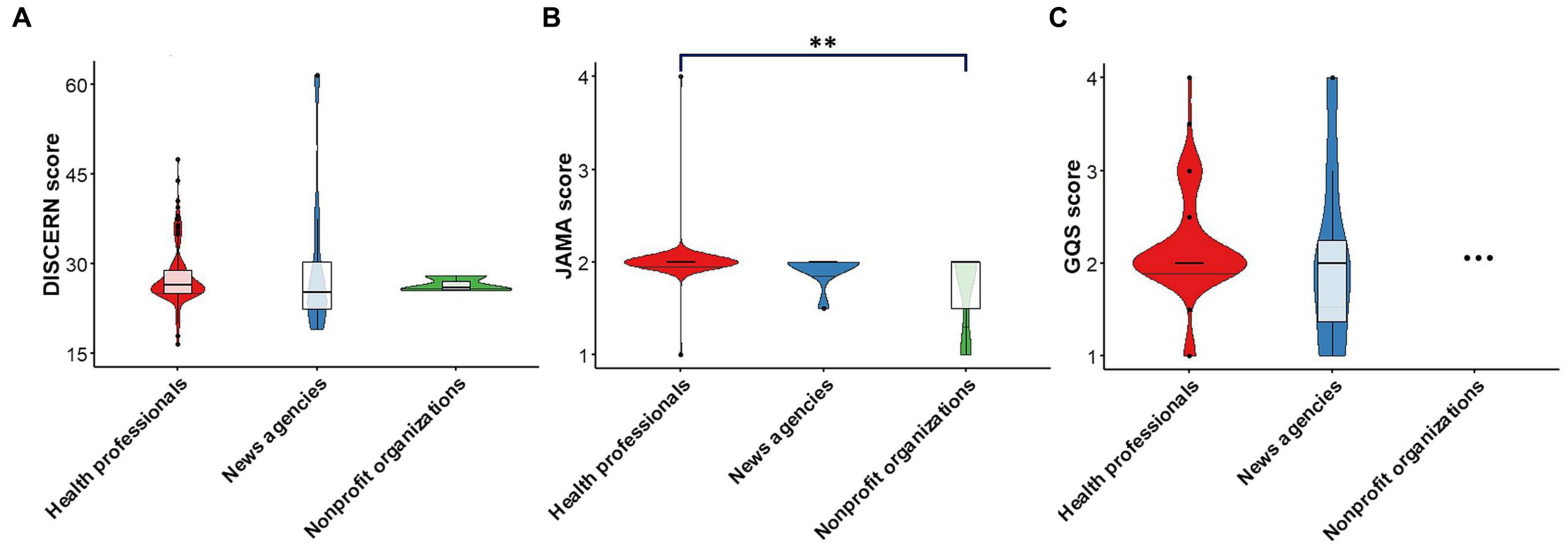
Comparisons of DISCERN scores, JAMA scores, and GQS scores among different sources. **(A)** Violin plots showing the total DISCERN scores among videos from different sources. **(B)** Violin plots showing the total JAMA scores among videos from different sources. **(C)** Violin plots showing the total GQS scores among videos from different sources. **P < 0.01.

Regarding the reliability of video publications, the median score for all videos was 16.00 (IQR 16.00–17.00). For the quality of treatment choices depicted in the videos, the median score for all videos was 8.00 (IQR 7.00–9.50). The overall quality score and total scores were 2.00 (IQR 2.00–2.00) and 26.50 (IQR 25.00–28.75), respectively (Table 6). Further analysis revealed no significant difference in total scores among the different groups (Figure 3A). We also evaluated the general quality of each video using the JAMA and GQS scales. The median JAMA value for all videos was 2.00 (IQR 2.00–2.00), and the median GQS value was 2.00 (IQR 2.00–2.00) (Table 6). Moreover, the JAMA values of videos from health professionals were significantly higher than those from nonprofit organizations (*P* < 0.01, respectively) (Figure 3B). Further analysis revealed no significant difference in the GQS scores of the videos (Figure 3C).

**Table 6:** DISCERN scores, JAMA scores, and GQS scores of videos by source.

Source of videos	DISCERN				JAMA	GQS
	Publication reliability	Treatment choices	Overall quality	Total scores		
Health professionals	16.00 (16.00, 17.00)	8.00 (7.00, 9.50)	2.00 (2.00, 2.00)	26.50 (25.00, 29.00)	2.00 (2.00, 2.00)	2.00 (2.00, 2.00)
News agencies	14.50 (13.00, 18.00)	8.75 (7.75, 12.25)	2.00 (1.50, 2.50)	25.25 (22.00, 32.75)	2.00 (2.00, 2.00)	2.00 (1.25, 2.50)
Nonprofit organizations	16.00 (15.25, 16.50)	9.00 (8.50, 9.00)	2.00 (2.00, 2.00)	26.00 (25.75, 27.00)	2.00 (1.50, 2.00)	2.00 (2.00, 2.00)
Overall	16.00 (16.00, 17.00)	8.00 (7.00, 9.50)	2.00 (2.00, 2.00)	26.50 (25.00, 28.75)	2.00 (2.00, 2.00)	2.00 (2.00, 2.00)

### Correlation analysis

Spearman correlation analysis indicated certain correlations among the characteristics of the videos. Comprehensiveness of video content scores was positively correlated with video duration (*r* = 0.420, *P* < 0.001), number of comments (*r* = 0.195, *P* < 0.05), and number of shares (*r* = 0.174, *P* < 0.05). GQS scores were found to be negatively or positively correlated with the number of days since upload (*r* = −0.212, *P* < 0.05), and video duration (*r* = 0.442, *P* < 0.001) (Table 7).

**Table 7:** The relationship between variables and comprehensiveness of video content, DISCERN, JAMA, and GQS scores.

Variable and analysis	Comprehensiveness of video content		DISCERN		JAMA		GQS	
	*r* value	*P* value	*r* value	*P* value	*r* value	*P* value	*r* value	*P* value
Days since upload	−0.079	0.354	−0.159	0.059	−0.070	0.409	−0.212	0.012
Video duration	0.420	0.000	0.141	0.381	0.055	0.520	0.442	0.000
Number of likes	0.113	0.183	−0.045	0.599	−0.017	0.840	0.066	0.433
Number of comments	0.195	0.020	−0.081	0.340	−0.049	0.560	0.036	0.672
Number of collections	0.095	0.262	0.018	0.835	−0.025	0.771	0.135	0.111
Number of shares	0.174	0.039	0.008	0.928	−0.055	0.517	0.119	0.160

## Discussion

### Major findings

In this cross-sectional study, we analyzed the content of HF-related videos on TikTok, evaluating the content comprehensiveness and quality using the DISCERN, JAMA, and GQS instruments. TikTok has implemented and updated stringent certification rules to ensure user interests, information security, and reliability. Only certified institutions, including public hospitals, medical associations, and medical media, are allowed to post medical-related videos (33, 34). As a result, the majority of videos (92.2%) were posted by health professionals, with only one advertisement video found during the search phase. Despite these certification rules, the quality of the videos did not meet expectations.

### Overall video quality and correlation analysis

Our results indicated that only a few videos provided comprehensive coverage of all aspects of HF and offered appropriate and trustworthy recommendations. Furthermore, when health professionals were categorized, variations in the comprehensiveness of content were observed among different types of professionals. When assessed using the DISCERN, JAMA, and GQS scales, most videos did not receive high scores in terms of reliability and quality. Although videos posted by health professionals scored higher than those from nonprofit organizations in the JAMA assessment, no significant differences were observed in DISCERN and GQS scores among the groups.

Our findings revealed a positive correlation between video duration and content comprehensiveness, as well as a positive correlation between video duration and GQS scores. This is primarily because longer videos can provide more information, thus enhancing their quality (35). The number of likes, comments, collections, and shares reflect the popularity of a video (36). We found a positive correlation between the number of comments and content comprehensiveness, indicating that high-quality videos are more likely to gain popularity. Notably, the number of days since upload showed a negative correlation with GQS scores, suggesting continuous improvement in video quality over time (37).

### The potential of social media in health education

Poor treatment of underlying heart diseases is a common cause of HF development. Patients with HF experience a lower quality of life compared to individuals with other chronic diseases, attributed to severe symptoms, frequent hospitalizations, and reliance on emergency services (38). While effective HF treatment requires precise adjustments and monitoring, medical personnel cannot provide continuous guidance to patients. Therefore, patients and caregivers must develop self-care abilities for HF management. The European Society of Cardiology (ESC) has established guidelines for self-care in HF patients, emphasizing the importance of patient education on treatment adherence, lifestyle changes, symptom monitoring, and appropriate responses to deterioration (39). High-quality health education plays a crucial role in enhancing patients’ self-care abilities, facilitating their understanding of their condition, collaboration with healthcare providers, and assumption of responsibility for their care (21). Self-care education programs and discharge education using the teach-back method have shown positive results in improving symptom perception, self-care management, and self-efficacy among HF patients (40, 41).

Patients, especially those with high morbidity and mortality conditions like HF, often turn to the internet for disease self-care information due to limited access to medical resources (42). Videos are recognized as an accessible and impactful medium for presenting complex health information. Social media platforms, particularly those with visual content, have become important sources of information for patients and effective educational tools for healthcare practitioners. Video-based education has been shown to improve HF knowledge, self-care maintenance, and adherence to self-care behaviors (43, 44). This highlights the potential of social media platforms, including TikTok, in providing health education for diseases with high morbidity and mortality, such as HF. However, the quality of HF-related videos on TikTok remains inadequate, emphasizing the need for stricter video publishing standards and procedures to improve the quality of medical content.

While social media offers many advantages in health education, enhancing the quality of medical-related videos on these platforms is essential. Firstly, considering the complex nature of medical content, short videos may not effectively convey key information about HF. Lengthening the duration of videos can address this issue, but excessively long videos may lead to viewer impatience and disinterest, resulting in video skipping (45). Dividing longer videos into a series of shorter videos covering different aspects of the disease can be a solution. However, the lack of quick access to other parts of the series may hinder viewers from watching the entire series. Therefore, TikTok should encourage video producers to create series videos and optimize the platform’s user interface for easy navigation and discovery of the complete series. Secondly, the diagnosis and treatment of HF is complex and require comprehensive knowledge and continuous updates among cardiovascular health professionals (46). Videos should focus on improving patients’ self-care abilities rather than overly explaining tasks meant for medical personnel. This requires concise, easy-to-understand, and actionable video content, which places higher demands on video producers. Aligning video content with the Revised Self-Care of HF Index (47) and the European HF Self-care Behaviour scale (48) can better enhance patients’ self-care abilities. However, self-care involves disciplines that cardiovascular health professionals are not particularly good at, such as nutrition, psychology, and exercise (39). Hence, collaboration among a multidisciplinary team or assistance from video platforms may be necessary to ensure the production of high-quality videos (49). Thirdly, TikTok should consider introducing professional certification for video creators who produce medical content. This certification mark would enhance audience recognition of reliable and professional information, facilitating the dissemination of accurate medical information (20).

### Strengths, limitations, and future directions

This study is the first attempt to evaluate the quality and reliability of HF-related videos on TikTok using multiple assessment tools (content comprehensiveness, DISCERN, JAMA, and GQS). Our analysis also examines the correlation between video characteristics (likes, comments, collections, and shares) and video quality. However, there are limitations to consider. Firstly, our sample only includes videos uploaded on Chinese TikTok platforms, limiting the generalizability of our results to other languages (e.g., English) and platforms (e.g., Kwai). Despite the platform-specific focus on Chinese TikTok, our study draws parallels with research on YouTube atrial fibrillation videos, indicating consistently low video quality (50). Given the representative nature of atrial fibrillation as a common cardiovascular disease, we believe our findings may be applicable to other languages or platforms, such as international versions of TikTok and YouTube. Another limitation is the lack of standardized methods in the literature for evaluating the quality of patient health information videos on TikTok (51). For instance, some analyzed videos have good science-based content but lack evidence-based references. While intended for patients, the information is more suitable for healthcare professionals (52).

Internet-based health promotion has gained significant attention, prompting TikTok to implement policies to strengthen oversight of medical and health-related videos to ensure reliability and quality to some extent (53). However, there is currently no formal worldwide guideline specifically focused on health-promoting videos. Health practitioners should take the initiative to address this matter. Effective health-promoting videos should achieve a balance between scientific accuracy, popularity, duration, and ease of understanding (21). Therefore, medical professionals should receive training to produce easily comprehensible videos that include evidence-based information to help viewers understand professional terminology. Additionally, video platforms should enhance the professional review process for uploaded videos, in addition to the updated uploader authentication program. Superior regulatory agencies should develop unified management standards and policies for medical-related videos due to significant differences in rules and regulations across video platforms. Moreover, making video quality rating information readily available to viewers would be beneficial. This approach would assist viewers in discerning video quality and accessing accurate health education information. Lastly, to address the limited capabilities and time of individual video producers, video platforms could consider organizing collaborations among video producers skilled in different fields to produce relevant videos.

## Conclusion

In this study, we evaluated the information quality of 141 TikTok videos related to HF. The findings indicated that the quality of these videos is inadequate. There were no significant differences in content comprehensiveness, quality, and reliability, as assessed by DISCERN and GQS scores, among the groups except for videos from health professionals which scored higher than those from nonprofit organizations in JAMA assessments. Given the growing popularity of TikTok, it is crucial for the platform to develop policies that encourage video producers to prioritize the enhancement of patients’ self-care abilities to create high-quality medical videos.

## Data availability statement

The original contributions presented in the study are included in the article/Supplementary material, further inquiries can be directed to the corresponding authors.

## Author contributions

XG: Conceptualization, Data curation, Formal analysis, Investigation, Methodology, Software, Supervision, Validation, Visualization, Writing – original draft, Writing – review & editing. BD: Data curation, Writing – review & editing. LL: Data curation, Formal analysis, Validation, Writing – review & editing. DS: Validation, Writing – review & editing, Data curation. ZR: Validation, Writing – review & editing, Conceptualization, Methodology, Supervision, Investigation.
