# Supplementary material for: TikTok video as a health education source of information on heart failure in China: a content analysis
Source: Front Public Health. 2023 Dec 11;11:1315393. doi: 10.3389/fpubh.2023.1315393 (PMC10749320; doi:10.3389/fpubh.2023.1315393)
Supplement: Supplementary file 1 [file Table_1.DOCX]

**Supplementary Table 1: DISCERN quality criteria for assessing the publication reliability and treatment choices quality of video. (Scoring ranges from 1 to 5)**

| **Section 1 IS THE PUBLICATION RELIABLE?** | |
| --- | --- |
| 1 | Are the aims clear? |
| 2 | Does it achieve its aims? |
| 3 | Is it relevant? |
| 4 | Is it clear what sources of information were used to compile the publication (other than the author or producer)? |
| 5 | Is it clear when the information used or reported in the publication was produced? |
| 6 | Is it balanced and unbiased? |
| 7 | Does it provide details of additional sources of support and information? |
| 8 | Does it refer to areas of uncertainty? |
| **Section 2 HOW GOOD IS THE QUALITY OF INFORMATION ON TREATMENT CHOICES?** | |
| 9 | Does it describe how each treatment works? |
| 10 | Does it describe the benefits of each treatment? |
| 11 | Does it describe the risks of each treatment? |
| 12 | Does it describe what would happen if no treatment is used? |
| 13 | Does it describe how the treatment choices affect overall quality of life? |
| 14 | Is it clear that there may be more than one possible treatment choice? |
| 15 | Does it provide support for shared decision making? |
| **Section 3 OVERALL RATING OF THE PUBLICATION** | |
| 16 | Based on the answers to all of these questions, rate the overall quality of the publication as a source of information about treatment choices |

**Supplementary Table 2: The Journal of American Medical Association (JAMA) scoring (1 point for answer ‘yes’, 0 point for answer ‘no’)**

| **Criteria** | **Description** |
| --- | --- |
| Authorship | Authors and contributors, their affiliations, and relevant credentials should be provided. |
| Attribution | References and sources for all content should be listed clearly, and all relevant copyright information noted. |
| Currency | Website ownership should be prominently and fully disclosed, as should any sponsorship, advertising, underwriting, commercial funding arrangements or support, or potential conflicts of interest. |
| Disclosure | Dates that content was posted and updated should be indicated. |

**Supplementary Table 3: Global Quality Score (GQS) (Scoring ranges from 1 to 5)**

| **GQS Definition** | **Score** |
| --- | --- |
| Poor quality, poor flow of the video, most information missing, not at all useful for patients | 1 |
| Generally poor quality and poor flow, some information listed but many important topics missing, of very limited use to patients | 2 |
| Moderate quality, some important information is adequately discussed | 3 |
| Good quality good flow, most relevant information is covered, useful for patients | 4 |
| Excellent quality and flow, very useful for patients | 5 |
